# Macrophage function in obesity-induced inflammation and insulin resistance

**DOI:** 10.1007/s00424-017-1955-5

**Published:** 2017-02-23

**Authors:** Mario A. R. Lauterbach, F. Thomas Wunderlich

**Affiliations:** 1 Institute of Innate Immunity, University Hospital, University of Bonn, Sigmund Freud Str. 25, 53127 Bonn, Germany; 2 Institute for Genetics, Cologne Excellence Cluster on Cellular Stress Responses in Aging-associated Diseases (CECAD); Center for Endocrinology, Diabetes and Preventive Medicine (CEDP) Cologne, Max Planck Institute for Metabolism Research Cologne, University of Cologne, Gleueler Straße 50, 50931 Cologne, Germany

**Keywords:** Macrophage, Polarization, Metaflammation

## Abstract

The steadily increasing obesity epidemic affects currently 30% of western populations and is causative for numerous disorders. It has been demonstrated that immune cells such as macrophages reside in or infiltrate metabolic organs under obese conditions and cause the so-called low-grade inflammation or metaflammation that impairs insulin action thus leading to the development of insulin resistance. Here, we report on data that specifically address macrophage biology/physiology in obesity-induced inflammation and insulin resistance.

## Introduction

The steadily rising prevalence of obesity incorporates a major health issue because it is attended by fatal obesity-associated disorders including not only the development of type 2 diabetes and fatty liver diseases but also the rising incidence for certain cancer entities [7, 54]. In the first instance, obesity alters whole body metabolism that frequently results in insulin resistance [5]. Insulin is produced by the pancreatic beta cells in response to rising blood glucose levels, thereby leading to glucose uptake in insulin responsive organs. Excessive glucose is stored in the white adipose tissue (WAT) as lipids and in the liver as glycogen that can be converted back to glucose during fasting or periods of increased energy demands. All these metabolic processes and many more are controlled by insulin [93]. The actions of insulin are mediated via binding to the insulin receptor (IR) [5]. The IR and the homologous insulin-like growth factor 1 receptor (IGF1R) are receptor tyrosine kinases that use adaptor molecules for their downstream signaling [48]. These molecules belong to the insulin receptor substrate (IRS) family of proteins that upon engagement of the IR or IGF1R are tyrosine phosphorylated further leading to phosphatidylinositide 3-kinase (PI3K) and AKT activation. In diabetes patients, insulin action is impaired. While type 1 diabetes patients exhibit beta cell/insulin loss due to autoimmune reactions against the pancreas, type 2 diabetes develops as a consequence of insulin resistance that is frequent in obese patients. In obesity, the compromised glucose uptake into metabolic organs induces hyperglycemia in turn accelerating insulin production in beta cells. The excessive insulin production can partly compensate for decreased insulin sensitivity but progresses to increased beta cell mass and ultimately to beta cell death. How obesity facilitates the development of insulin resistance has been discovered over the last decade. Obesity has been accepted as low-grade inflammatory state that is also known as metaflammation [38]. Metaflammation is mainly derived from innate immune cells, e.g., macrophages whose derivation, fate, and functional consequences are discussed in this review.

## Tissue resident macrophages

Macrophages are cells of the innate immune system that populate every organ. They display great functional plasticity and are required for maintenance of tissue homeostasis, immunity against invading pathogens, and tissue repair. Different organs harbor different specialized tissue resident macrophages, which include red pulp and marginal zone macrophages in the spleen, microglia in the brain, peritoneal macrophages, osteoclasts in the bone, alveolar macrophages in the lung, and the two major metabolic tissue macrophage subsets in the liver and adipose tissue—liver Kupffer cells and adipose tissue macrophages, respectively [73]. Certain tissue macrophage subsets populate their organs during embryogenesis, while in adulthood, tissue resident macrophage subsets are replenished by monocytes that are recruited from the bone marrow [22]. The two major subsets of monocytes are termed inflammatory and patrolling monocytes. They are distinguished by a defined panel of surface markers and chemokine receptors and have distinct chemotactic properties. Inflammatory monocytes are C-C chemokine receptor type 2 (CCR2) and lymphocyte antigen 6 c (Ly6C) positive, whereas patrolling monocytes are CCR2 and Ly6C negative, but express high levels of CX3 chemokine receptor 1 (CX3CR1). Expression of these different chemokine receptors allows them to follow diverging chemokine gradients. Under steady state, patrolling monocytes crawl along the vasculature where they function as immune sentinels. They can enter tissues through expression of CX3CR1 and differentiate into tissue resident cells with dendritic cell- and macrophage-like features [21, 86]. The degree of macrophage turnover under homeostatic conditions varies between organs [22]. Inflammatory monocytes are recruited to tissues in response to infection or tissue damage via chemokine (C-C motif) ligand 2 (CCL-2). In the absence of inflammation, they remain in the blood circulation [21].

## Macrophage ontogeny

Precursors of several tissue macrophage subsets emerge during embryogenesis. Hematopoiesis during embryonic development occurs at two sites in two stages—first extra-embryonically in the yolk sac and later on in the fetal liver [22]. Hematopoiesis in the yolk sac was termed “primitive” and thought to solely preserve macrophage pools during embryogenesis, while tissue resident macrophages are derived from adult hematopoietic stem cells (HSC). However, fate-mapping studies revealed that numerous tissue resident macrophage populations are of prenatal origin and emerge from the yolk sac or the fetal liver. Microglia for example were shown to originate from the yolk sac, while Langerhans cells are mainly of fetal liver origin [23]. The use of (Myb-deficient) mice, which lack the bone marrow hematopoietic stem cell compartment, shed more light on the development of tissue resident macrophages [96]. These mice still develop tissue resident macrophage populations including Langerhans cells, Kupffer cells, microglia, lung alveolar, splenic red pulp, and peritoneal macrophages, indicating that yolk sac-derived progenitors give rise to several long-lasting tissue macrophage subsets beyond embryonic development [124]. Notably, these macrophage populations are maintained during adulthood under homeostatic conditions owing to their self-renewal potential.

It is an ongoing debate whether determination to the distinct tissue resident macrophage subsets occurs during embryonic development or is induced by signals from the local environment once macrophage populate their final tissue. On the one hand, some findings support the concept that macrophage precursors could already be committed to give rise to a certain subset by the time they enter the circulation to populate their target organ. Recent studies for example suggested that microglia are derived from a distinct yolk sac precursor, while other tissue resident macrophages derive from embryonic hematopoietic precursors [36, 98]. Other studies in contrast provided evidence that cues from the local tissue environment are crucial for differentiation into the distinct tissue resident macrophage subsets. By comparing different tissue resident macrophage populations, it has been assessed that tissue-specific factors shape the chromatin and enhancer landscape of macrophages thus enabling the transcription of subset-specific genes [27, 59]. Retinoic acid for example drives the differentiation of peritoneal macrophages by inducing the expression of the transcription factor GATA-6, which in turn activates a peritoneal macrophage-specific transcriptional program that is crucial for maintenance and functionality of these cells [81, 90]. The idea of in situ differentiation is supported by findings that tissue resident macrophages originate from a common erythro-myeloid progenitor (EMP) in the yolk sac, which populates the fetal liver before entering the blood stream to give rise to tissue resident macrophages [25]. Using single-cell RNA sequencing, a recent study investigated the chronology of macrophage differentiation during embryogenesis. EMPs give rise to premature macrophages that share a common gene expression signature. Induction of specific expression profiles is initiated during organogenesis when pre-mature macrophages enter the tissue [65].

## Polarization states of macrophages

Tissue resident macrophages express a multitude of pattern recognition receptors (PRRs) that enable them to sense a wide range of microbial molecules and danger signals. Upon pathogen encounter, they induce a cascade that signals the quality of infection or danger, induces an inflammatory state, and recruits other immune cells to the site of infection [44]. Together with neutrophils, they produce bactericidal molecules and phagocytose pathogens to terminate the infection. After the infection has been cleared, they resolute inflammation by anti-inflammatory cytokines and lipid mediators and govern tissue repair by phagocytosing debris and promoting regeneration of extracellular matrix [73].

Macrophages exhibit a high degree of functional plasticity, and the nature of an inflammatory trigger as well as the local cytokine milieu will determine the respective macrophage polarization and its functional state. In this regard, early on, the distinction between classically activated and alternatively activated or—in analogy to the Th1/Th2 nomenclature of T cells—M1 and M2 macrophages has been made [64]. In vitro, these subsets can be induced by incubation with interferon gamma (IFNγ) and lipopolysaccharide (LPS) or interleukin-4 (IL-4), respectively. The M1/M2 nomenclature was in particular used to classify macrophages into cells with pro-inflammatory or anti-inflammatory properties or certain effector functions. The following studies revealed that distinct pro- or anti-inflammatory stimuli elicit distinct transcriptomic profiles, which led to the proposal of the spectrum model or more recently the multidimensional model [24]. However, many important findings were made using the aforementioned stimuli. Also, many classical M1 and M2 markers are still used to assess the functional state of macrophages in vivo. We thus will refer to macrophages with pro-inflammatory properties as M_pro_ and macrophages with anti-inflammatory properties as M_anti_.

Triggers like the Th1 cytokine IFNγ and/or toll-like receptor (TLR) ligands such as LPS initiate a pro-inflammatory response that equips macrophages to fight bacterial infections [64]. These stimuli activate signaling cascades that result in a global transcriptional reprogramming. In this context, signal transducer and activator of transcription (STAT) 1 and 2 and nuclear factor kappa-light-chain-enhancer of activated B cells (NF-κB) are key transcription factors of IFNγ and TLR signaling, respectively [60]. Pro-inflammatory macrophages (M_pro_) generally have inflammatory properties and are crucial for fighting bacterial infections and immunity against tumors. However, excessive activation can also result in tissue damage and autoimmunity. They present antigen and produce bactericidal agents such as reactive oxygen species (ROS) and nitric oxide (NO). The latter is synthesized by inducible nitric oxide synthase (iNOS) from arginine. They furthermore secrete a multitude of inflammatory cytokines including tumor necrosis factor alpha (TNFα), IL1-β, IL-6, IL-12, and IL-18 [63].

Three major pro-inflammatory cytokines are TNFα, Il-1β, and IL-6. TNFα is one of the first cytokines secreted by macrophages during infection and crucially involved in septic shock. It activates the vascular endothelium and initiates the acute phase in the liver. IL-6 also activates the acute phase and induces fever. In addition, it acts on lymphocytes and activates cytotoxic cells or stimulates differentiation of plasma cells. Depending on the signaling pathways that are activated upon receptor binding, IL-6 can also have anti-inflammatory properties [68]. IL-1β is a strong pyrogen, but in addition can induce the secretion of prostaglandins in the central nervous system. Notably, IL-1β and TNFα are both potent inducer of IL-6 and thereby amplify the inflammatory cascade [17]. IL-12 induces Th1 differentiation and together with IL-18 induces IFNγ production by Th1 and natural killer (NK) cells which in turn acts on macrophages in a feed forward loop [63].

Alternatively activated macrophages (M_anti_) were initially described during helminth infections and exhibit an anti-inflammatory phenotype. In vitro, they can be induced by the Th2 cytokines IL-4 and IL-13. Similarly as described for pro-inflammatory stimuli, binding of IL-4 to its receptor will result in a global transcriptional reprogramming. One of the key transcription factors mediating these changes is STAT6. It acts in concert with peroxisome proliferator-activated receptor gamma coactivator 1-alpha (PGC-1α) and peroxisome proliferator-activated receptor gamma and delta (PPARγ and PPARδ) which, as will be discussed below, are particularly involved in reprogramming cellular energy metabolism [60]. M_anti_ are important players during helminth infection, response to tissue damage, resolution of inflammation, and wound healing, but can also foster fibrosis and tumor growth [26]. Their most important effector molecules include arginase, lectins, scavenger receptors and the cytokines IL-10 and the IL-1 receptor antagonist (IL1-RA). Arginase converts arginine to ornithine, which in turn is used during tissue repair for polyamine and collagen synthesis. Scavenger receptors and lectins mediate clearance of debris and apoptotic cells during resolution of inflammation, while IL-10 and IL-1RA are potent suppressors of inflammation [26, 63].

## Macrophage metabolism

The differential use of arginine was an early indication that pro- and anti-inflammatory triggers induce diverging metabolic changes in macrophages. Over the last years, a series of discoveries have highlighted a tight linkage between cellular metabolism and macrophage effector functions [83]. While distinct metabolic features of many immune cells were described, we are just beginning to understand how nutrient availability can shape immune responses. In the following, we will briefly summarize these findings with a focus on glycolysis, β-oxidation, and amino acid metabolism.

Glycolysis describes the sequential breakdown of glucose to pyruvate, which is either converted to lactate and secreted or imported into mitochondria. When shuttled into mitochondria, pyruvate is converted to acetyl-coenzyme A (CoA), which enters the tricarboxylic acid (TCA) cycle by condensation with oxaloacetate. The TCA cycle generates NADH and FADH2, which are used to generate ATP via oxidative phosphorylation (OXPHOS). Apart from glycolysis, multiple catabolic pathways converge into the TCA cycle such as β-oxidation and glutaminolysis. β-Oxidation of fatty acids generates acetyl-CoA, while glutamine can enter the TCA by sequential conversion to α-ketoglutarate [15]. Apart from its central role in catabolism, the TCA cycle also serves as a metabolic hub that can redirect its intermediates for anabolic reactions when required. Citrate for example can be exported from mitochondria and cleaved to acetyl-CoA and oxaloacetate by ATP-citrate lyase. Acetyl-CoA in turn serves as a precursor for fatty acid or cholesterol biosynthesis and is substrate for acetylation reactions in the cytoplasm and the nucleus. OXPHOS is the most effective way for cells to generate ATP and is used by most quiescent cell types to cover their energetic demands [15]. OXPHOS, however, requires oxygen and has rather slow ATP generation kinetics. Thus, under hypoxic conditions or conditions of increased ATP demand, cells switch from OXPHOS to glycolysis to generate ATP. This phenomenon—glycolytic activity under normoxic conditions—is termed Warburg effect after its discoverer Otto Warburg. Initially discovered in cancer cells, proliferating cells or cells with high anabolic demands such as the developing embryo, epithelial cells, or activated immune cells exhibit Warburg metabolism [108]. Switching to glycolysis does not only allow for fast ATP generation, but also enables cells to diverge glycolytic intermediates into anabolic pathways such as amino acid, lipid, or nucleotide biosynthesis, instead of oxidizing them via TCA and OXPHOS [108].

Increased glycolysis was described early on in LPS-stimulated macrophages [83]. A series of recent studies has underpinned that commitment to Warburg metabolism equips macrophages to fulfill their effector functions such as production of ROS or NO, phagocytosis, and secretion of inflammatory mediators in the context of bacterial infection. Upon activation with pro-inflammatory stimuli like LPS or IFNγ, macrophages undergo metabolic reprogramming and exhibit increased rates of glycolysis and decreased OXPHOS. By diverting ATP generation from OXPHOS to glycolysis, mitochondria are available for ROS production [115]. Furthermore, instead of being used for ATP production, citrate is exported from mitochondria and used for fatty acid biosynthesis [42]. To compensate for decreased conversion of citrate to α-ketoglutarate, glutamine is funneled into the TCA via anaplerosis to α-ketoglutarate [102]. The molecular mechanisms driving these changes are just being uncovered. Some are NF-κB dependent: LPS for example induces PFKFB3, which increases glycolytic flux by generating fructose-2,6-bisphosphate [91]. Moreover, the NF-κB responsive gene hypoxia-inducible factor 1 alpha (HIF1α) was identified as another central metabolic regulator in response to LPS [12]. HIF1α is an essential mediator of the hypoxic response, partly by promoting a shift from OXPHOS to glycolysis. Under normoxic conditions, it is constantly degraded by the proteasome and only stabilized under hypoxic conditions. Ubiquitination and subsequent degradation of HIF1α are initiated by hydroxylation of proline residues by prolyl hydroxylases (PHDs). In certain cancer cells that exhibit Warburg metabolism, inhibition of PHDs can lead to HIF1α stabilization under normoxic conditions [97]. A similar effect was observed in response to LPS stimulation. In this context, ROS, succinate accumulation and iron were shown to contribute to PHD inhibition [76, 99, 102]. While ROS and succinate inhibit PHD activity, PHDs require iron as cofactor for the hydroxylation reaction. Indeed, ferritin decreases iron availability to PHDs after LPS stimulation and thereby downregulates PHD-dependent HIF1α hydroxylation [99]. While HIF1α sustains proliferation in cancer cells [97], it upregulates glycolysis [12], and also specifically boosts IL-1β expression [102] in response to LPS stimulation. As described before, arginine metabolism to citrulline and NO is a key effector function of pro-inflammatory macrophages. Indeed, changes in glutamine metabolism upon stimulation supply arginine and boost NO production by iNOS [47].

While LPS-activated macrophages mainly rely on glycolysis, IL-4-activated macrophages exhibit an oxidative phenotype [47]. Upon IL-4 stimulation, macrophages increase fatty acid uptake, β-oxidation, and OXPHOS [109]. These metabolic rearrangements are initiated by STAT6 and PGC-1β. In concert with PPARγ and PPARδ, they induce mitochondrial biogenesis and expression of genes that are involved in β-oxidation and OXPHOS [79]. These studies also highlighted a role for PPARs in the control of metabolic disease and maintaining insulin sensitivity. Furthermore, recent studies have highlighted the tight connection between macrophage metabolism and its effector functions in helminth infection and identified lysosomal lipolysis as alternative pathway to foster β-oxidation. Notably, the authors of this study showed that blockade of lysosomal liposysis during *H. polygyrus* infection results in defective clearance of the pathogen and inhibits commitment to OXPHOS by macrophages [41]. IL-4-stimulated macrophages maintain an intact active TCA cycle and generate ATP mainly via OXPHOS. While changes in β-oxidation are the most striking metabolic adaptations in response to IL-4, metabolomic studies also revealed that glycolysis as well as glutaminolysis contribute to TCA activity [47]. Notably, glutamine deprivation inhibits M_anti_ macrophage induction in vitro [47], whereas inhibition of glycolysis only affects a small subset of IL-4 target genes [11]. Thus, while macrophages undergo drastic metabolic changes in response to pro- and anti-inflammatory triggers, like LPS and IL-4, respectively, their diverse effector functions and locations differentially affect whole body metabolism underlining their central role in organismal physiology.

## Adipose tissue macrophages and Kupffer cells in homeostasis

Besides their role in sensing infection and tissue damage, tissue resident macrophages have important homeostatic and trophic functions. Limiting inflammation and maintaining tissue homeostasis are extra crucial functions of the liver resident Kupffer cells (KC) and adipose tissue macrophages (ATM). In obesity, insulin resistance develops as a consequence of metaflammation in which elevated circulating levels of pro-inflammatory cytokines such as TNFα and IL-6 negatively affect the insulin signaling cascade [37]. The main source for these inflammatory mediators in obesity is hepatic and WAT macrophages [122]. Macrophages adapt in their residing tissue to local circumstances and exert numerous effector functions such as phagocytosis and cytokine production. In the obese state, macrophages in the WAT and the liver are major players in regulating metaflammation. Macrophages sense factors derived from pathogens or from cells belonging to innate and adaptive systems as well as from specialized cells in the affected tissue. We will refer here on the impact of ATM and liver-derived KC in the development of obesity-associated insulin resistance.

## Adipose tissue macrophages

Adipose tissue is one of the major metabolic organs that stores excess nutrients as triacylglycerides and releases fatty acids in the fasted state, which serve as energy source for peripheral tissues. Under homeostatic conditions, adipose tissue is populated with macrophages that exhibit a M_anti_ like phenotype and govern adipocyte lipid metabolism by secreting factors such as IL-10 and catecholamines. IL-10 enhances adipocyte insulin sensitivity and lipogenesis [62], whereas catecholamines trigger lipolysis in adipocytes [75]. Under conditions of excessive lipolysis, they control release of fatty acids into the circulation by serving as buffer [55]. While the ontogeny of other tissue macrophage subsets is well studied, less is known about ATM. Under inflammatory conditions, monocytes enter adipose tissue in a CCR2-dependent manner [62]. The origin of ATMs under homeostatic conditions is a matter of debate. Interestingly, WAT contains a pool of c-Kit^+^/Lin^−^/Sca-1^+^ cells that share features of hematopoietic stem cells [10]. This population fails to populate bone marrow in non-irradiated mice, but is capable of replenishing the innate immune cell pool in adipose tissue [85]. ATMs might thus be regenerated in situ independent of the bone marrow.

A pioneering study from Hotamisligil and Spiegelman identified adipocytes as source of TNFα in the WAT that ultimately impaired insulin signaling in obesity [39]. However, findings by Xu et al. demonstrated that mainly the stromal vascular fraction of the obese WAT expresses inflammatory cytokines [122]. Currently, the view that the majority of other cells than adipocytes in the obese WAT are macrophages is supported, whereas in lean conditions, these cells represent approximately 10% [112]. While in lean WAT, mainly alternative M_anti_ like macrophages express anti-inflammatory molecules, in obese, WAT macrophage polarization is shifted towards a pro-inflammatory M_pro_ like phenotype. The increased abundance and activation of macrophages in the obese WAT can be accounted by adipose tissue stress that includes elevated amounts of free fatty acids and LPS [28]. LPS, which is presumably microbiome derived, is not only abundant in WAT, but also found in the circulation of obese individuals [8]. LPS and fatty acids such as palmitate activate TLR4 signaling in ATMs that polarizes them towards M_pro_ macrophages [13, 50]. Subsequently, these stimuli trigger expression of TNFα and IL-6 in ATMs that compromise insulin action not only locally in the WAT, but also systemically since they are released to circulation [82]. Thus, it is tempting to speculate that metaflammation is a consequence of local innate immune response in the WAT that spills over via the blood to other organs due to the blood soluble factors involved. Of note, caloric restriction-induced weight loss including improvements in systemic insulin sensitivity and whole body glucose metabolism ameliorated metaflammation in the liver but not in adipose tissue suggesting that long-lived ATMs maintain WAT inflammation [95].

The M_anti_ like ATMs in lean WAT express the cell surface marker CD206 and exhibit anti-inflammatory properties such as IL-10 expression. These M_anti_ like ATMs synergize with regulatory T cells (Treg) and innate type 2 lymphoid cells (ILC2) to maintain the anti-inflammatory WAT environment [18, 70]. Tregs, T cells, ILC2 cells, and even adipocytes provide anti-inflammatory IL-4, IL-13, and IL-33 in the lean WAT to keep ATMs in an M_anti_ like state [49, 117]. In the course of obesity, monocyte recruitment as well as local proliferation gives rise to novel ATMs that polarize towards a pro-inflammatory M_pro_ like phenotype that express the surface marker CD11c [51, 62, 113]. Abruptly, the WAT environment has changed from anti- to pro-inflammatory conditions indicated by the lack of Treg cells and infiltration of cytotoxic and Th1 T cells as well as NK cells [78, 114]. Besides the already mentioned M_pro_ polarizing LPS, T cells and NK cells in the obese WAT provide IFNγ, which sustains polarization towards the M_pro_ phenotype [114]. ATMs accumulate intracellular lipids not only via phagocytosis of dying adipocytes resulting in crown like structures in the WAT, but also via fatty acid transporter-mediated uptake [123]. The metabolism of obese ATMs has changed to glycolysis, which is necessary for the production of nitric oxide by iNos to increase pro-inflammatory macrophage responses [20]. M_pro_ macrophages take up glucose via glucose transporter 1 that further triggers M_pro_ polarization [19].

Therefore, it is not surprising that the polarizing environment in the obese WAT in vivo cannot be completely recapitulated in vitro in bone marrow-derived macrophages (BMDM) [123]. For instance, upregulation of CD11c expression is not induced by sole LPS and IFNγ treatment in BMDM, but can be restored by coculture with adipocytes [56]. Treatment with high glucose, insulin, and palmitate induces a M_pro_ like phenotype in BMDM culture that releases inflammatory cytokines [56]. Consistently, insulin receptor inactivation in macrophages prevents M_pro_ like polarization [66].

Conversely, M_anti_ macrophages in vitro similarly as present in the lean WAT have not been reported, but M_anti_ can be differentiated in BMDM cultures by several means. Supplementation of culture media with IL-4 or IL-13 creates different CD206 expressing M_anti_ macrophages, than those that require TLR and IL1R agonists [61, 100]. Another M_anti_ population can be differentiated by IL-10 which shares anti-inflammatory properties with the other two BMDM subtypes [72]. Strikingly, reactivity to IL-6 is required to polarize towards all of these M_anti_ type macrophages. In particular, arginase 1 and IL-4Rα expression critically depend on IL-6 signaling [67]. Taken together, reallocation from M_pro_ like towards M_anti_ type ATMs might be a promising strategy to resume whole body insulin sensitivity that would prevent fatal diseases associated with obesity such as development of metabolic syndrome and the progression to cancer. In line with this evidence, nematode infection or vaccination with nematode antigens reprograms the obese WAT microenvironment towards anti-inflammatory conditions resulting in improved insulin sensitivity and glucose tolerance [4].

## Kupffer cells and liver infiltrating macrophages

The liver is essential for life due to its metabolic as well as immunoregulatory functions. On the one hand, under high energy conditions and hyperglycemia, hepatocytes in the liver import excessive glucose that is converted to glycogen. During fasting periods, the liver maintains blood glucose levels via hepatic glucose production that includes degradation of glycogen by glycogenolysis and breakdown of proteins and lipids through gluconeogenesis upon prolonged fasting. On the other, the liver is the first line defense against pathogens via the acute phase response and clears infected as well as exhausted cells. In line with its immune function, the blood stream entering the liver through the portal vein runs through the gut as well as the WAT before. In liver sinusoids, specialized liver resident macrophages, the KC, sense and combat invading commensals from the gut to prevent spreading along circulation [88]. Gut-derived LPS for example can be detected in portal vein but less in circulation [45]. In obesity, impaired storage of excessive lipids in the WAT leads to liver fat accumulation resulting in steatosis and fatty liver diseases [107]. The inappropriate fat storage in the liver results in lipotoxicity which in turn leads to liver damage and inflammation [74, 118]. Thus, in obesity, lipotoxicity and elevated microbial load from the microbiota result in excessive inflammation mediated by KCs and infiltrating macrophages. Interestingly, depletion of phagocytic cells in the liver via clodronate liposomes prevents steatosis, inflammation, and the development of insulin resistance thereby identifying hepatic macrophages as mediators of obesity-associated pathologies [58]. Hepatic macrophages crosstalk to liver non-parenchymal cells and adapt their polarization to states of liver condition. Obesity-induced pro-inflammatory cytokines and LPS polarize KC towards M_pro_ that in turn induce a vicious cycle of TNFα, IL-6, and IL-1β that further boosts and deteriorates liver functions [46, 53]. The inflammatory boost in obesity does not alter KC numbers but dramatically increases infiltration of CCR2-positive monocytes [52, 71]. Furthermore, inflammatory TNFα released by hepatic macrophages limits systemic insulin action, and IL-6 signaling in hepatocytes instructs downregulation of the inflammatory response in hepatic macrophages [120]. Collectively, inflammatory signaling in the liver differentially affects hepatic cell types and might result in complicating outcomes in whole body metabolism.

## Obesity-induced low-grade inflammation and insulin resistance

Obesity contributes to the development of insulin resistance through the so-called obesity-associated low-grade inflammation or metaflammation. Over the course of this process, immune cells infiltrate metabolic organs, mainly WAT and liver, where they secrete pro-inflammatory cytokines that act locally but also systemically after being released into circulation [82].

The cytokine levels in obesity do not reach levels upon infection, but instead are elevated 2–3-fold compared to homeostatic conditions. Moreover, while during infection, pro-inflammatory cytokines increase acutely and stagnate with the elimination of the pathogen, the obesity-associated low-grade inflammation exhibits chronic character suggesting that dynamic modes of action have to be taken into account. The best-studied inflammatory players in obesity are TNFα and IL-6, but also include IL-17, CCL-2, and many others. In this paragraph, we will delineate how TNFα- and IL-6-induced signaling impact on the insulin signaling cascade.

## TNFα

In a ground breaking report, Hotamisligil and colleagues discovered that the obese WAT contains high levels of the pro-inflammatory cytokine TNFα [39]. In a follow-up study, they could show in tissue culture experiments that media supplemented with TNFα impaired insulin action [40]. On a molecular level, TNFα compromises activating tyrosine phosphorylations in the insulin signaling cascade mainly of IRS molecules, but also the IR. While at that time, adipocytes were believed to be the source of TNFα in obesity, Xu et al. demonstrated that the stromal vascular fraction of WAT secretes pro-inflammatory cytokines that inhibit insulin signaling [122]. Moreover, bone marrow transplantation experiments revealed that mainly macrophages are the source of TNFα in the obese WAT [14]. TNFα interferes with insulin recpetor signaling at the level of IRS molecules. IRS molecules are phosphorylated on inhibitory serine residues by TNFα-induced kinases such as IκB kinase (IKK), c-Jun N-terminal kinase (JNK), and atypical protein kinase C (aPKC) thereby preventing further downstream signaling [77]. Of note, these kinases have redundant as well as individual functions in IRS phosphorylation and point mutations of IRS1 serine residues to non-phosporylatable counterparts yielded the conflicting result that mutant mice developed insulin resistance [9]. Nevertheless, genetic mouse models provided novel insight into how TNFα-induced signaling interferes with insulin signaling in obesity. On the one hand, TNFα knockout mice exhibit normal glucose tolerance when exposed to normal food, but are protected from the development of obesity-induced insulin resistance in the absence of body weight gain alterations on the other [106]. While this study demonstrated the critical importance of TNFα in the development of insulin resistance, the dissection of further downstream signaling at the TNFα-induced kinase level has revealed surprising results. TNFα induces a dual kinase system that comprises the IKK complex and the JNK kinases [101]. The IKK complex contains the kinases IKK-1 and IKK-2 as well as the NFκB essential modulator NEMO, all of which are essential for mouse viability as revealed by knockout studies. Muscle-specific IKK-2 inactivation showed no effect on diet-induced obesity and alterations in glucose homeostasis [89]. However, while hepatic IKK-2 inactivation conferred insulin sensitivity in this organ, but not in muscle and WAT, myeloid IKK-2 inactivation resulted in systemic improvements of insulin sensitivity upon high-fat diet (HFD) challenge mainly due to reduced inflammatory cytokine release [1, 6]. Otherwise, hepatic NEMO inactivation resulted in global improvements in insulin sensitivity under obese conditions, but in contrast to IKK-2 KO mice, these mice developed liver tumors due to ongoing TNFα-induced cell death and compensatory hyperproliferation [119]. Thus, though activated by the same upstream stimulus, kinases may play redundant and non-redundant roles in impairing the actions of insulin.

In contrast to the IKK complex genes, knockout of one of the three individual JNK kinases (JNK-1, JNK-2, and JNK-3) is well tolerated in mice, whereas double knockout for the most abundant peripheral JNK-1 and JNK-2 is embryonic lethal [57]. It has been shown that JNK-1 but not JNK-2 knockout mice are protected against obesity-induced impairments of glucose homeostasis suggesting an essential role for JNK-1 in serine phosphorylation of IRS molecules [35]. However, conditional mouse models aimed at unraveling the cell type-specific as well as redundant functions of the JNK genes in the development of obesity-associated insulin resistance. Opposite to what was expected, hepatic inactivation of JNK-1 revealed a modestly impaired glucose tolerance and hepatic lipid accumulation suggesting a function of JNK-1 in the prevention of steatosis and liver fat accumulation [110]. Moreover, skeletal muscle-specific JNK-1 deficiency revealed a minor role in glucose metabolism [84], whereas WAT-specific JNK-1 deletion decreased obesity-induced IL-6 levels and thus ameliorated diet-induced insulin resistance [92]. However, neuronal-specific JNK-1 deficiency most closely resembled the phenotype of complete JNK-1 knockout mice indicating that a redundancy between JNK isoforms in peripheral organs exist [3]. In line with these findings, JNK-1/JNK-2 double-deficient macrophages are unable to produce inflammatory cytokines, and thus, mice with macrophage-specific deletion of JNK-1 and 2 are protected against obesity-induced disorders [30, 31]. Collectively, deciphering organ-specific downstream actions of TNFα in obesity-induced insulin resistance revealed redundant as well as non-redundant kinase functions on inhibitory IRS serine phosphorylation.

## IL-6

IL-6 is a pleiotropic cytokine that plays crucial roles in metabolic and immune cells. Similar to TNFα, IL-6 is also slightly increased in serum of obese individuals and mice, which is believed to be detrimental for metabolism [2]. Here, a bulk of IL-6 is produced by the stromal vascular fraction of visceral fat depots, which is directly delivered to the liver via the portal vein [92]. In contrast, IL-6 is increased manifold during intense exercise in muscle (regulated by JNK-1) that provides beneficial effects on metabolism [116]. IL-6 exerts its function by binding to the IL-6 receptor α chain (IL-6Rα) and the GP130 signaling chain complex in classical membrane-bound pathway. The IL-6Rα is expressed mainly on hepatocytes and immune cells, but also non-IL-6Rα-expressing cells can be rendered IL-6-responsive by a mechanism called trans-signaling [33, 94]. IL-6 trans-signaling is the process where adam proteases cleave/shed the IL-6Rα from the surface of IL-6Rα-expressing cells that when bound to serum IL-6 generates the soluble IL-6Rα (sIL-6Rα). sIL-6Rα in turn binds ubiquitously expressed GP130 on cells not expressing IL-6Rα to activate the same signaling cascade as the classical membrane IL-6Rα signaling [94]. Both cascades initiate Janus kinase (JAK)2/STAT-3-dependent transcriptional activation of target genes such as SOCS-3 [33]. SOCS-3 is not only a negative regulator of IL-6 signaling but also inhibits insulin signal transduction at the IRS protein level. Here, IL-6-induced SOCS-3 leads to ubiquitination and subsequent proteasomal degradation of IRS1 [104, 105]. Consistently, clinical studies link obesity-induced insulin resistance with increased IL-6 levels. Importantly, weight loss reduces circulating IL-6 and improves insulin sensitivity [2]. On the other hand, however, IL-6 itself provides beneficial effects on metabolic processes such as regulation of hepatic gluconeogenesis indicating that the molecular mechanism of how IL-6 affects metabolism and insulin sensitivity is not completely understood [43, 87]. When IL-6 would exert negative effects on glucose metabolism exclusively, the expectation for IL-6 knockout mice would be the maintenance of insulin sensitivity. However, while Di Gregorio et al. did not observe metabolic alterations in IL-6 knockout mice [16], Wallenius and colleagues demonstrated that IL-6 inactivation favors the development of mature onset obesity and diabetes implicating that IL-6 action on metabolism might be even more complex than hitherto assumed [111].

A potential aspect that may explain these differences might be the chronic/constant presence of IL-6 under obesity conditions. We have demonstrated that in diet-induced obesity, the chronically high IL-6 levels lead to the development of hepatic IL-6 resistance [29]. IL-6 resistance is caused by basal IL-6-activated STAT3 that chronically increases expression of SOCS-3 [121]. SOCS-3 in turn inhibits IL-6 receptor signaling, which can be identified by the inability of liver cells to react with STAT3 phosphorylation upon exogenous IL-6 treatment. Such high hepatic SOCS-3 levels might not only have impact on IL-6 signaling, but also on the insulin receptor signaling cascade by interfering with IRS proteins. Consistently, inactivation of SOCS-3 in hepatocytes improves hepatic insulin action and steatosis in young mice, but at older age, these mice develop obesity and insulin resistance due to the activation of acute phase response and overt inflammation [103]. IL-6 signaling in hepatocytes therefore somehow crosstalks with liver resident KCs that are the source for the inflammatory response. In line with this evidence, inactivation of the IL-6 receptor in hepatocytes fulminates in the development of systemic insulin resistance as a consequence of KC-mediated inflammation. Thus, IL-6 signaling in hepatocytes controls whole body insulin sensitivity by limiting KC-mediated inflammation [120]. Therefore, considering the differential aspects of IL-6 action under lean and obese conditions will contribute to our molecular knowledge how the low-grade metaflammation impacts on insulin signaling to ultimately result in the development of metabolic disorders. Given that IL-6 not only impacts on metabolism but also on the development of cancer and that obesity increases the incidence of cancer entities with an inflammatory microenvironment, the context-specific dissection of signaling cascades will be necessary for the development of novel therapeutic interventions to combat such fatal obesity-associated diseases [68].

## Conclusion/outlook

Inflammation triggered by macrophages constitutes a turning point in the development of obesity-related insulin resistance. It is not only that mediators secreted by macrophages trigger insulin resistance, at the same time, also beneficial effects exerted by ATM and KCs under homeostasis are compromised. These include maintenance of a local anti-inflammatory milieu, insulin sensitivity, and control of lipolysis and energy expenditure. Research over the last 15 years has uncovered mechanisms that drive changes in macrophage polarization and how molecules secreted by macrophages affect lipid metabolism and insulin receptor signaling in metabolic organs. The use of mouse models pointed out that macrophage polarization in adipose tissue and the liver is critical for development and progression of metabolic disease. Reprogramming from inflammatory M_pro_ towards alternative M_anti_ macrophages might represent a promising strategy to recover whole body insulin sensitivity that would prevent fatal diseases associated with obesity. Indeed, thiazolidinediones and omega 3 fatty acids are such exemplary drugs or dietary factors that improve metabolic disease partly by dampening macrophage-mediated inflammation [34, 80]. In this context, targeting immune cell metabolism might also hold great potential. Systemic insulin resistance is also observed during pregnancy or puberty, but most notably also during infection, in particular sepsis. During sepsis, inflammatory cytokines in particular IL-6 and TNFα induce a state of insulin resistance [69]. Moreover, while in type 2 diabetes, lipolysis in adipose tissue and gluconeogenesis in the liver slowly emerge as a result of insulin resistance, inflammatory cytokines actively induce lipolysis in adipose tissue, protein catabolism in the muscle, and gluconeogenesis in the liver and muscle during sepsis. Central mediators include glucagon, epinephrine, and cortisol, which are released as a consequence of the inflammatory cascade [32]. From an evolutionary point of view, this metabolic response serves to support the immune system in fighting the infection and limiting its spread throughout the body in the context of sepsis. As outlined above M_pro_ macrophages and also other immune cells rely on glycolysis to fuel their inflammatory response to fight infection [83]. In the context of type 2 diabetes and obesity, increased glucose availability to immune cells might initiate a feed forward loop that fosters inflammation and further aggravates disease.
